# Mitochondrial Costs of Being Hot: Effects of Acute Thermal Change on Liver Bioenergetics in Toads (*Bufo bufo*)

**DOI:** 10.3389/fphys.2020.00153

**Published:** 2020-03-11

**Authors:** Damien Roussel, Yann Voituron

**Affiliations:** CNRS, UMR 5023, Laboratoire d’Ecologie des Hydrosystèmes Naturels et Anthropisés, Université Claude Bernard Lyon 1, Lyon, France

**Keywords:** H_2_O_2_ release, oxygen consumption, ATP synthesis, amphibian, temperature, mitochondrial efficiency

## Abstract

Global climatic warming is predicted to drive extreme thermal events, especially in temperate terrestrial environments. Hence, describing how physiological parameters are affected by acute temperature changes would allow us to understand the energy management of organisms facing such non-predictable and constraining events. As mitochondria play a key role in the conversion of energy from food into ATP but also produce harmful reactive oxygen species, the understanding of its functioning is crucial to determine the proximal causes of potential decline in an animal’s performance. Here we studied the effects of acute temperature changes (between 20 and 30°C) on mitochondrial respiration, ATP synthesis rate, oxidative phosphorylation efficiency (ATP/O), and H_2_O_2_ generation in isolated liver mitochondria of a terrestrial ectotherm, the common toad (*Bufo bufo*). Using succinate as the respiratory substrate, we found that the mitochondrial rates of oxygen consumption, ATP synthesis, and H_2_O_2_ generation increased as the temperature increased, being 65, 52, and 66% higher at 30°C than at 20°C, respectively. We also found that the mitochondrial coupling efficiency (ATP/O) decreased, while the oxidative cost of ATP production (H_2_O_2_/ATP ratio) increased. The present results further indicate that between 40 and 60% of temperature effects on mitochondrial ATP production and H_2_O_2_ generation was *at minima* driven by an action on the oxidative capacity of the mitochondria. These results suggest that *B. bufo* may need to allocate extra energy to maintain ATP production and protect cells from oxidative stress, reducing the energy allocable performances.

## Introduction

Long-term records in climatic data indicate that the global surface temperature has increased by 0.2°C per decade in the past 30 years (Intergovernmental Panel On Climate Change [IPCC], 2014). However, it is important to differentiate between “climate” which is observed year after year and “weather” that is much less predictable. Predictions of ever more extreme weather patterns are associated with climate change, especially in terrestrial environments, such as higher frequency and duration of heat waves and heavy rainfall events during the summer in North America and Europe (Fischer and Knutti, 2013; Ummenhofer and Meehl, 2017). Both theoretical predictions and experimental data show that extreme weather events impact biodiversity and shift the geographic species distribution (Bellard et al., 2012). In addition to the anthropic use of land and water, which can induce rapid and stochastic environmental changes, weather (not climate) will probably be the most challenging characteristic of climate change facing terrestrial animals. Freshwater ecosystems will also be strongly impacted by extreme events with floods or drought-induced variation of temperature, pollution, and oxygen content (Xia et al., 2015). The aquatic populations must behaviorally and/or physiologically respond to optimize the performance of individuals under these new conditions (Woodward et al., 2016).

Temperature is one of the most prominent abiotic parameters. Environmental temperature governs the body temperature of ectothermic animals which in turn determines their biochemical reactions, cellular metabolism, and physiological functions. Thus, the thermal dependence of biological activities and performance puts ectothermic organisms at risk due to the rise in extreme thermal events driven by global climatic warming, even if major drivers of extinction are hard to predict (Kearney et al., 2009). In most animals, including ectothermic species, aerobic metabolism is fundamentally important in supplying the energy that is needed for cellular activity, performance, and animal life. By providing most of the ATP needed for cellular activities, the mitochondria constitute the main physiological link between environmental resources and animal performance. Reactive oxygen species (ROS) are also inevitable by-products of mitochondrial aerobic metabolism, a biochemical process that occurs when electrons leak from the electron transport chain during oxidative phosphorylation to directly react with molecular oxygen (Brand, 2016). Thus, a key reason for focusing on mitochondrial functioning is its importance in supplying energy and regulating the oxidative balance that are involved in many life history traits and trade-offs (Monaghan et al., 2009; Seebacher et al., 2010; Salin et al., 2015; Schulte, 2015). Mitochondrial oxidative phosphorylation and ROS generation are known to be directly influenced by temperature (Cassuto, 1971; Abele et al., 2002; Heise et al., 2003; Chamberlin, 2004; Paital and Chainy, 2014; Chung and Schulte, 2015; Wiens et al., 2017), generating similar shapes of performance curves with those of many biochemical and physiological processes (Pörtner et al., 2007; Schulte, 2015). Performance increases as temperatures rise, followed by a plateau when the processes reach thermal optimum, followed by a steep decline at higher temperatures (Pörtner et al., 2007; Schulte, 2015). Therefore, understanding the mitochondrial responses in these organisms during extreme events may be crucial to determine the proximal causes of potential population decline (Heller and Zavaleta, 2009).

An increase in mitochondrial activity during the first ascendant phase of the thermal performance curves would be associated with an increased output of cellular energy in the form of ATP and thus would be beneficial for animal performance. However, such beneficial effect of warming on mitochondrial activity will be counterbalanced by an increase in energy cost associated with the maintenance of membrane potential due to increased membrane fluidity (Dufour et al., 1996; Chamberlin, 2004; Brown et al., 2012), which would decrease the mitochondrial coupling efficiency (Monternier et al., 2014). The thermal effects on mitochondrial ROS generation can also be compared: (i) it can be increased following an increase in mitochondrial oxidative activity, as discussed in the free radical theory of aging (Barja, 2013) or (ii) it can be decreased following increased membrane fluidity as suggested by the “uncoupling to survive” hypothesis (Brand, 2000). In the present paper, we deciphered the mitochondrial mechanisms involved in an acute (without acclimation) increase of temperature in an ectotherm: the common toad (*Bufo bufo*). The aim of this work was to understand how oxygen consumed by the mitochondria was allocated between the maintenance (proton leakage, H_2_O_2_ generation) and productive (ATP synthesis) costs of mitochondrial energy metabolism.

## Materials and Methods

### Animals

We chose the common toad as a model since it is one of the most common amphibians in Europe having a wide latitudinal and altitudinal distribution (Borkin and Veith, 1997) as well as a large variation in body temperature. The body temperatures range from 12 to 19°C at night and from 28 to 34°C when in daytime retreats or abroad (Meek and Jolley, 2006). Fifteen male toads (*B. bufo*) were collected in the spring (March) from a pond located 40 km south of Lyon, France (4°92 E, 45°50’ N). The breeding number was estimated to be over 3,500 males. The animals were individually housed in a box maintained at 20°C for 1 week. The mean body mass (±SD) was 39.9 ± 3.1 g at the time of the experiments.

### Mitochondrial Isolation

Mitochondria were isolated in an ice-cold isolation buffer (250 mM sucrose, 1 mM EGTA, and 20 mM Tris-HCl, pH 7.4) from three livers per preparation, with all steps performed at 4°C (Salin et al., 2012; Roussel et al., 2015). A total of five independent mitochondrial preparations were done in the present study. Briefly, 2–3 g of liver (mean tissue mass was 0.93 ± 0.06 g per animal) was homogenized with a Potter–Elvehjem homogenizer (three passages). The homogenate was centrifuged at 800 × *g* for 10 min. The resulting supernatant was centrifuged at 1,000 × *g* for 10 min, filtered through cheesecloth, and re-centrifuged at 8,700 × *g* for 10 min to pellet mitochondria. The liver mitochondrial pellet was washed twice by suspension in the isolation buffer and centrifuged at 8,700 × *g* for 10 min. The protein concentration of the mitochondrial suspension was determined by the biuret method with bovine serum albumin as a standard. The toad mitochondrial preparations contained a dark pigment which absorbed at 540 nm, and the absorbance of the same volume of mitochondria in water containing 0.6% deoxycholate and 3% NaOH was subtracted.

### Mitochondrial ATP Synthesis and Oxidative Phosphorylation Efficiency

Liver mitochondria (1.5 mg/ml) were incubated in 500 μM respiratory medium (120 mM KCl, 1 mM EGTA, 5 mM KH_2_PO_4_, 2 mM MgCl_2_, 0.3% of essentially free fatty acid bovine serum albumin, and 3 mM HEPES adjusted to pH 7.4) supplemented with glucose (20 mM) and hexokinase (1.5 U/ml). The air-saturated medium was assumed to contain 521, 479, and 437 nmol of O/ml at 20, 25, and 30°C, respectively. Oxygen consumption and ATP synthesis rates were performed at 20, 25, or 30°C using succinate (5 mM) as respiratory substrate in the presence of rotenone (5 μM). The mitochondrial ATP synthesis was initiated by the addition of 100, 25, or 10 μM and followed by glucose-6-phosphate accumulation using an ATP-regenerating system (hexokinase plus glucose) as previously described for frogs (Salin et al., 2012; Roussel et al., 2015). Briefly, after recording the phosphorylating respiration rate, four 100-μl samples of mitochondrial suspension were withdrawn from the suspension every 2 min and immediately quenched in perchloric acid solution (10% HClO_4_ and 25 mM EDTA). After centrifugation of the denatured protein and neutralization of the resulting supernatant, the glucose-6-phosphate content of the samples was measured by spectrophotometry according to Lang and Michal (1974).

The maximal oxidative activity of the electron transport system (ETS) was measured with a Clark oxygen electrode (Rank Brother Ltd., United Kingdom) in a stirred and closed chamber with a volume of 500 μl, thermostatically controlled at three different temperatures (20, 25, or 30°C). The maximal fully uncoupled respiration rate, associated with the maximal ETS activity, was measured on mitochondria respiring on succinate (5 mM) in a respiratory buffer supplemented with 5 μM rotenone, 2 μg/ml oligomycin, and 2 μM carbonyl cyanide *p*-tri-fluoro-methoxy-phenyl-hydrazone (FCCP).

### Mitochondrial Radical Oxygen Species Production

The measurement of mitochondrial H_2_O_2_ generation was performed with a Kontron fluorometer (model SFM-25) in a stirred chamber of 1 ml volume, thermostatically controlled at three different temperatures (20, 25, or 30°C). Liver mitochondria (0.4 mg/ml) were incubated in a respiratory medium supplemented with 5 U/ml horseradish peroxidase and 1 μM Amplex red. The rate of mitochondrial H_2_O_2_ release was assessed following the linear increase in fluorescence (λ_excitation_ = 560 nm and λ_*emission*_ = 584 nm) in the presence of succinate (5 mM) and then after the addition of ADP (100 μM). The fluorescent signal was calibrated using a standard curve prepared with known concentrations of H_2_O_2_.

### Thermal Sensitivity of Mitochondrial Metabolism (Q_10_)

The temperature coefficient Q_10_ was calculated for mitochondrial fluxes (oxygen consumption, ATP synthesis, and H_2_O_2_ generation) using the following formula:

$${\text{Q}}_{10}={\left(R_{2}/R_{1}\right)}^{10/\left(T_{2}-T_{1}\right)}$$

where R_1_ and R_2_ denote the mitochondrial flux at higher (T_2_) or lower (T_1_) temperatures, respectively. The Q_10_ value was calculated for the temperature range of 20–25 and 25–30°C.

### Statistical Analyses

The results are presented as mean ± SEM. A one-way repeated-measure analysis of variances (RM ANOVA) was performed to test the temperature effect on fluxes (oxygen consumption, ATP production, and H_2_O_2_ release) as well as free electron leak. Multiple linear regression was performed to test the relationships between the different parameters, with temperature as an independent variable. When variances in homogenization and/or homoscedasticity were not observed, non-parametric Friedman test was used. The statistical analyses were performed using JMP 12 (SAS Institute Inc., Cary, NC, United States). A 5% (*p* = 0.05) level of significance was used in all of the tests.

## Results

### ATP Synthesis and Oxidative Phosphorylation Efficiency

**FIGURE 1:**
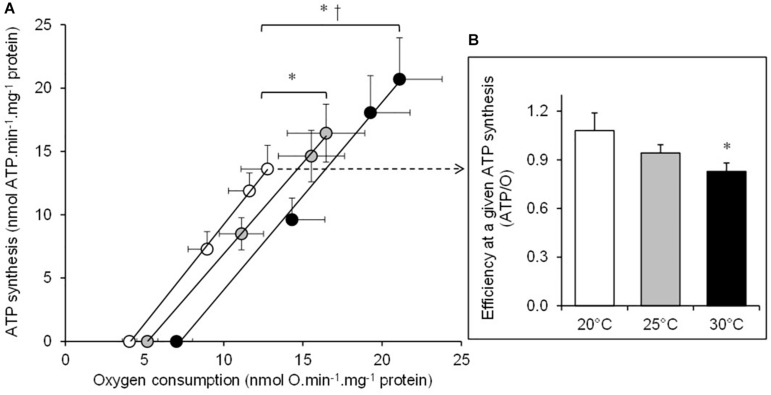
The effect of temperature on the mitochondrial oxidative phosphorylating efficiency. **(A)** The rates of ATP synthesis and oxygen consumption were determined at 20°C (white circles), at 25°C (gray circles), and at 30°C (black circles) in the mitochondria isolated from the liver of toad and respiring on succinate. Mitochondrial fluxes were titrated with increasing concentrations of ADP in the presence of glucose and hexokinase (see section “Materials and Methods” for more details). Values are means ± SEM for *N* = 5 independent mitochondrial preparations. **(B)** The coupling efficiency calculated at the highest common ATP synthesis rate between the three temperatures (13.6 nmol ATP min^–1^ mg^–1^ protein). Shortly, oxygen consumption rates were calculated at this highest common ATP synthesis rate by using individual linear relation curves. Then, the resulting effective ATP/O ratios were calculated at 20°C (white bars), at 25°C (gray bars), and at 30°C (black bars). The values are means ± SEM for *N* = 5 independent mitochondrial preparations. * and † mean significantly different from 20 and 25°C, respectively (*p* < 0.05).

**FIGURE 2:**
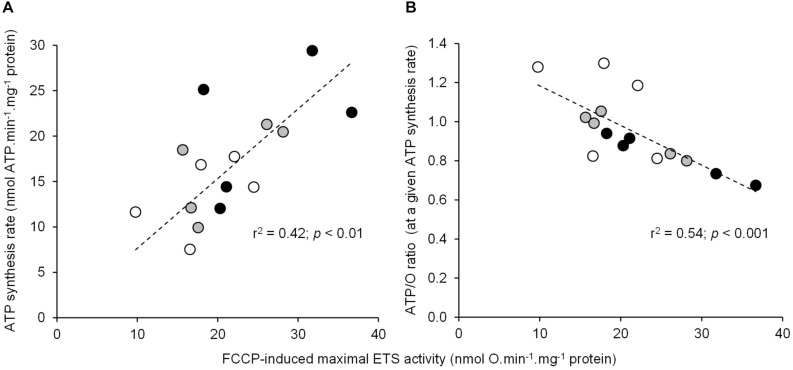
Relationship between the individual maximal oxidative activities and the ATP synthesis rate or coupling efficiency. The correlation of mitochondrial FCCP-induced maximal ETS activity using succinate as the respiratory substrate with **(A)** the maximal rate of ATP synthesis (mean values are reported in Table 1) or **(B)** the coupling efficiency (ATP/O ratio) calculated at the highest common ATP synthesis rate (mean values are shown in Figure 1B). Individual values show five independent mitochondrial preparations from the liver of toad measured at 20°C (white circles), at 25°C (gray circles), and at 30°C (black circles).

The basal non-phosphorylating rates of oxygen consumption increased with increasing temperature (basal state; Table 1). The maximal rates of ATP synthesis and corresponding oxygen consumption increased from 20 to 25 and to 30°C (active state; Table 1). The respiratory control ratio (RCR) was not significantly affected by temperatures (RCR_20_°_*C*_ = 3.14 ± 0.18; RCR_25_°_*C*_ = 3.15 ± 0.14; RCR_30_°_*C*_ = 3.05 ± 0.21). Figure 1 shows the effect of temperature on the linear relationship between the rates of ATP synthesis and of oxygen consumption in mitochondria working at different steady-state rates. There was no significant effect of temperature on the slope values (ATP/O_20_°_*C*_ = 1.53 ± 0.13; ATP/O_25_°_*C*_ = 1.47 ± 0.07; ATP/O_30_°_*C*_ = 1.47 ± 0.11), indicating that these were parallel relations. Since the basal non-phosphorylating respiration rates (the intercepts with the *x*-axis) were significantly affected by temperature (basal state; Table 1), the linear relations were significantly shifted to the right as the temperature increased (Figure 1). Consequently, more oxygen was consumed at any steady-state rates of ATP production when the temperature increased, indicating that mitochondrial coupling efficiency decreased with increasing temperature. This is clearly illustrated in Figure 1B where the coupling efficiency was calculated at the maximal rate of ATP synthesis measured at 20°C, the highest common mitochondrial ATP production between thermal conditions. Interestingly, there was a close correlation between FCCP-induced maximal activity of ETS and the maximal rate of ATP synthesis and efficiency, indicating that at least 40% of the temperature effects on mitochondrial ATP production and efficiency were driven by an action on the oxidative capacity of the mitochondria (Figure 2).

**TABLE 1:** Effect of temperature on mitochondrial metabolism and H_2_O_2_ production.

	Temperature (°C)			Statistical analysis	
		
	20	25	30	*F*-values	*P*-values
**Oxygen consumption rate (nmol O min ^–1^ mg protein ^–1^)**					
Basal state	3.90.4	5.20.7*	7.01.0^†^	15.7	
Active state	12.81.7	16.52.5*	21.12.7^†^	38.2	
Maximal ETS	18.22.5	20.92.6	25.63.6^†^	9.6	
**ATP synthesis rate (nmol ATP min^–1^ mg protein^–1^)**					
Active state	13.61.9	16.42.3*	20.73.3^†^	15.2	*p* < 0.01
**H_2_O_2_ generation rate (pmol H_2_O_2_ min^–1^ mg protein^–1^)**					
Basal state	13316	17817*	24024^†^	38.8	*p* < 0.0001
Active state	738	939*	1558^†^	255.5	*p* < 0.0001
**Free electron leak (%H_2_O_2_/O)**					
Basal state	3.30.2	3.50.2	3.50.3	n.s.	n.s.
Active state	0.590.04	0.600.06	0.770.08^†^	8.5	*p* < 0.05

The mitochondrial oxygen consumption rates and the H_2_O_2_ production were measured in isolated liver mitochondria fueled with succinate alone (basal state) or with ADP (active state) or with FCCP in the presence of oligomycin (maximal ETS, maximal activity of the electron transport system). Free electron leak is the percent of oxygen turned into H_2_O_2_ instead of H_2_O (%H_2_O_2_/O). The values are expressed as means ± SEM (*N* = 5). *p < 0.05 versus 20°C; ^†^*p* < 0.05 versus 25°C.

### Mitochondrial H_2_O_2_ Generation and Oxidative Cost of ATP Synthesis

**FIGURE 3:**
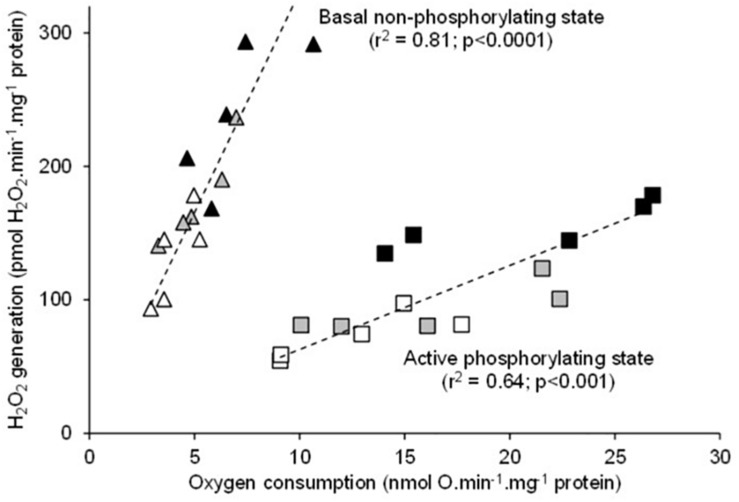
Relationship between the individual H_2_O_2_ production and the mitochondrial oxygen consumption rates during fueling with succinate alone (basal non-phosphorylating state) or with succinate and ADP (active phosphorylating state). The individual values show five independent mitochondrial preparations from the liver of toad measured at 20°C (white circles), at 25°C (gray circles), and at 30°C (black circles).

**FIGURE 4:**
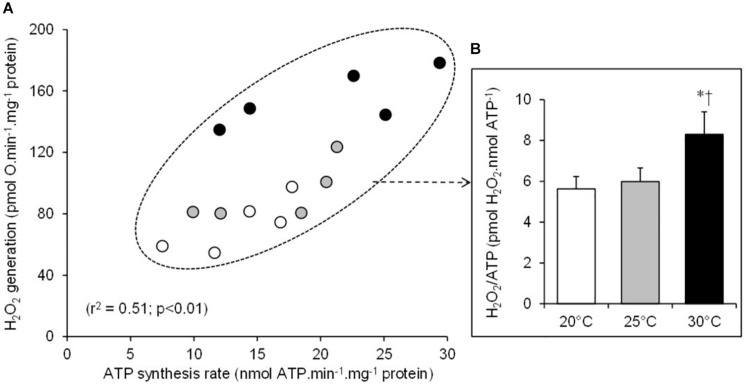
Relationship between the individual H_2_O_2_ production and the mitochondrial ATP synthesis rates **(A)**. The individual values show five independent mitochondrial preparations from the liver of toad measured at 20°C (white circles), at 25°C (gray circles), and at 30°C (black circles). **(B)** The oxidative cost of ATP production (% H_2_O_2_/ATP ratio) calculated at 20°C (white bars), at 25°C (gray bars), and at 30°C (black bars). The values are means ± SEM for *N* = 5 independent mitochondrial preparations. * and † mean significantly different from 20 and 25°C, respectively (*p* < 0.05).

There was a significant positive relationship between oxygen consumption and H_2_O_2_ generation in the mitochondria respiring on succinate under both basal non-phosphorylating and active phosphorylating states (Figure 3). The higher the respiration rates, the higher the H_2_O_2_ production. Hence, the mean values of H_2_O_2_ generation significantly increased between 20 and 25 and to 30°C under both basal and active states (Table 1). The electron leak (%H_2_O_2_/O) was not affected by temperatures in the basal non-phosphorylating state. The electron leak was not significantly changed between 20 and 25°C but was significantly higher at 30°C than at 20 and 25°C in the active phosphorylating state. There was also a significant positive relationship between H_2_O_2_ generation and ATP synthesis rates (Figure 4A) with temperature effect (values at 30°C are significantly higher than those at 20 and 25°C). This temperature effect explains the higher oxidative cost of ATP production (H_2_O_2_/ATP ratio) at 30°C compared with those at 20 and 25°C (Figure 4B).

### Thermal Sensitivity of Mitochondrial Metabolism and H_2_O_2_ Production

**FIGURE 5:**
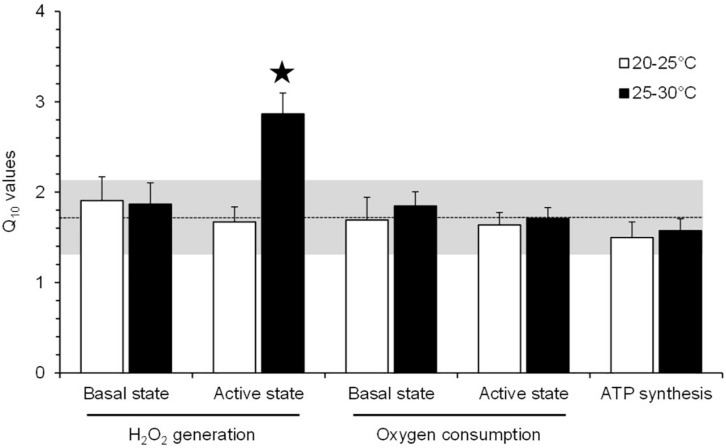
Q_10_ values for the effect of acute temperature change on the mitochondrial parameters of toad liver. The values are means ± SEM for *N* = 5 independent mitochondrial preparations. *Significantly different from all of the other Q_10_ values (*p* < 0.05). The dashed line represents the mean of Q_10_ values for all mitochondrial parameters (±SD is represented by the horizontal gray-shaded rectangle), excluding the Q_10__(__25__–__30_°_*C*__)_ value for the H_2_O_2_ production under an active phosphorylating state.

Thermal sensitivity (Q_10_) was not significantly different for any aspect of mitochondrial function, except for H_2_O_2_ production under the active phosphorylating state (Figure 5). Mitochondrial H_2_O_2_ production under the active state exhibited a 1.7-fold increase in Q_10_ value compared with the values of other mitochondrial parameters (Figure 5).

## Discussion

The data presented here show that the rapid increase in temperature induces higher mitochondrial rates of oxygen consumption, ATP synthesis, and H_2_O_2_ production. These results are in accordance with the acute temperature effects on oxidative phosphorylation activity and ROS production reported in several ectothermic species (Cassuto, 1971; Abele et al., 2002; Heise et al., 2003; Paital and Chainy, 2014; Chung and Schulte, 2015; Wiens et al., 2017). However, high temperatures also decrease the mitochondrial coupling efficiency (ATP/O). This result indicates that the beneficial effect of increasing temperature on mitochondrial ATP synthesis is associated with an energy cost mediated by a loss of efficiency. These results indicate that the mitochondrial energy transduction system has to consume more oxygen (+ 20 ± 11% at 25°C and + 37 ± 13% at 30°C) so as to oxidize more substrates to sustain the production of a given amount of ATP. The negative impact of temperature on mitochondrial coupling efficiency is likely explained by a thermal effect on the fluidity of the inner membrane and a subsequent increase in proton permeability at high temperature (Chamberlin, 2004; Brown et al., 2007, 2012; Monternier et al., 2014; Power et al., 2014; Chung and Schulte, 2015; Jarmuszkiewicz et al., 2015; Zoladz et al., 2016). Nevertheless, our data also indicate that the loss of efficiency is also partly ascribed to a thermal stimulation of the activity of the ETS (Figure 2B). This result is in agreement with the previously reported negative link between the mitochondrial oxidative capacities and the efficiency of oxidative phosphorylation (Nogueira et al., 2001; Romestaing et al., 2008; Roussel et al., 2018). Mechanistically, an increased activity of the ETS would build up a higher membrane potential, triggering an increase in the voltage-dependent proton leakage and its negative control over the effective coupling efficiency (Brand et al., 1993; Roussel et al., 2004; Brand and Nicholls, 2011). Notwithstanding the underlying mechanisms, the present data highlight an extra energy need to generate cellular energy during hot weather events, suggesting that the potential energy allocated to performance such as growth or reproduction will be more limited. These elements provide a cellular mechanism of the “metabolic meltdown” phenomenon in ectotherms recently developed by Huey and Kingsolver (2019).

The H_2_O_2_ generation in toad liver mitochondria is also positively correlated to the rates of oxygen consumption, with these two mitochondrial fluxes increasing as temperature increases. The increase of H_2_O_2_ production along with respiratory activity in response to an acute rise in temperature has been widely reported in mitochondria from ectothermic species (Abele et al., 2002; Heise et al., 2003; Paital and Chainy, 2014; Chung and Schulte, 2015; Wiens et al., 2017). In the present study, we used succinate, a FADH_2_-linked substrate, which drives ROS production which is critically sensitive to proton-motive force (Korshunov et al., 1997; Miwa et al., 2003; Keller et al., 2004). As previously stated, the inner membrane proton leakage increases with increasing temperature, which should have led to a decreased inner membrane potential and thus ROS production. The present data have not verified this mechanism. This could be explained by the fact that the proton leak is not the only process to change with temperature; the activity of the electron transport chain also changes. These observations suggest that the thermal effect on the activity of the substrate oxidation system, which builds up membrane potential, compensates for the one on the proton leak, which consumes membrane potential. This is indirectly supported by the absence of thermal effect on the RCR. The RCR referred to the ratio between the active phosphorylating respiration rate, which is controlled by the activity of ATP turnover and substrate oxidation, and the basal non-phosphorylating respiration rate, which is mainly controlled by the activity of proton leak (Brand and Nicholls, 2011). Hence, no changes in RCR indicate that the activities of both the oxidative phosphorylation and the proton leak were similarly affected by temperature. This hypothesis is even more directly supported by data reporting no major decrease of membrane potential values with increasing temperatures in the mitochondria from several ectothermic species (Chamberlin, 2004; Trzcionka et al., 2008; Chung and Schulte, 2015). Thus, the thermal increase in H_2_O_2_ production reported here was mainly driven by an increased electron flow through the ETS since the fractional electron leak (%H_2_O_2_/O ratio) was not altered by temperatures, at least in basal state and in active state at 20 and 25°C. However, an oxidative cost of ATP production (H_2_O_2_/ATP) appears at the highest temperature tested (30°C) when the mitochondria were functioning at their maximal phosphorylating rate. This thermal sensitivity of H_2_O_2_ production was explained, at least in part, by a significant increase in free electron leakage (%H_2_O_2_/O ratio) during the maximal phosphorylating activity. On the whole and notwithstanding the underlying mechanisms, such thermal effects would clearly represent an oxidative cost of the mitochondrial functioning in ectotherms facing an extremely hot weather event. Of note, the stability of RCR highlights that oxidative stress occurred well before high temperatures cause a failure in mitochondrial activity.

**FIGURE 6:**
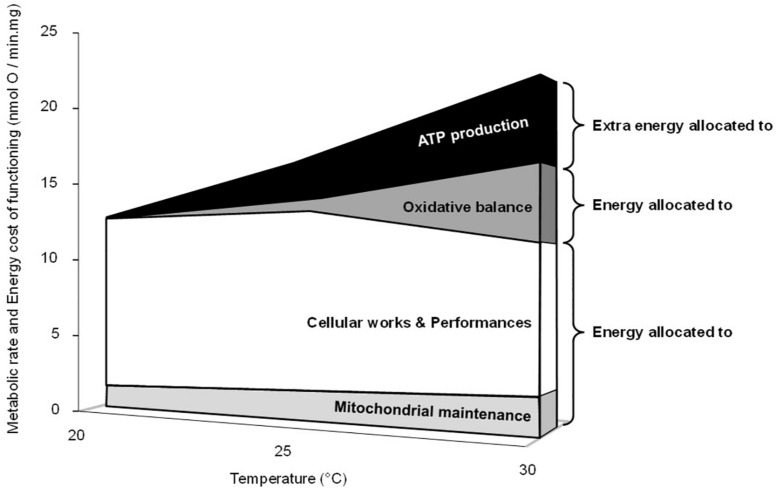
Synoptic summary of the effects of acute temperature change on mitochondrial metabolism and energy costs. The upper limit in the figure represents the positive thermal effect on the maximal phosphorylating oxygen consumption rates (active states in Table 1), which is linearly related to the rates of ATP synthesis (Figure 1A). The “extra energy allocated to ATP synthesis” is related to the thermal decrease of the coupling efficiency and represents the extra oxygen needed to produce a given amount of ATP. It was calculated by using the percent changes in effective coupling efficiency measured at 25 and 30°C compared with values at 20°C (-15 and -22%, respectively; Figure 1B). The “energy allocated to oxidative balance,” the “oxidative cost,” is related to the thermal increase in H_2_O_2_ generation. It was calculated by using the mean value of the differences between the ratios%H_2_O_2_/O and%H_2_O_2_/ATP measured in the phosphorylating active state at 25°C or at 30°C and values measured at 20°C (+7 and +33%, respectively; Table 1 and Figure 4B). “Mitochondrial maintenance” represents the need for oxygen and substrate consumption to counteract proton leakage and maintain the mitochondrial membrane potential value in active state. Even though the mitochondrial proton leakage is low in the phosphorylating active state, it still occurs, representing between 10 and 15% of the maximal phosphorylating oxygen consumption rate in liver mitochondria (Brand et al., 1993; Roussel et al., 2004, 2018). Here we used the fix value of 11%, arguing that the thermal increase in membrane conductance and subsequent proton leakage was counterbalanced by the thermal increase in the activity of the electron transport system (Table 1).

Figure 6 presents a synoptic summary of the subcellular benefits and the costs of acute temperature increase on liver oxidative metabolism in the common toad. At the mitochondrial level, the rate of ATP synthesis (the power of life) increased in the range of the temperature tested, which represents a clear beneficial effect of temperature. However, this beneficial effect was counteracted by at least two energy costs. The first cost is the decrease of mitochondrial efficiency, which implies extra oxygen consumption (the fuel for life) and so a higher quantity of substrate to produce a given amount of cellular energy in the form of ATP. At the level of an organism, this would drive an increased foraging activity to fulfill resource needs to maintain ATP homeostasis and animal performance. Hence, part of the initial benefits would have to be invested into locomotor activity. The second cost is the elevated H_2_O_2_ production and especially the increase in the oxidative cost of ATP production (H_2_O_2_/ATP ratio) at 30°C. This cost would trigger increased oxidative damage to cellular macromolecules. Hence, part of the initial energy benefits would have to be invested into oxidative defenses and/or repair systems to counteract these increased oxidative damages. After summing up the two main costs, the loss of efficiency and the rise in H_2_O_2_ production, the energy costs associated with warming become higher than the initial benefit of having increased metabolic power at elevated temperatures. Indeed the energy left over to perform cellular activity becomes lower at 30°C than at 20 or 25°C (Figure 6). Interestingly, the increase of H_2_O_2_ production along with the decrease in coupling efficiency in response to an acute rise in temperatures has been reported in mitochondria from different ectothermic tissues (Abele et al., 2002; Heise et al., 2003; Paital and Chainy, 2014). Nevertheless, it must be kept in mind that Figure 6 only summarized the patterns observed at the level of the liver mitochondria. Although these results might preclude alteration in the liver energy homeostasis in a warm environment, they must be taken with caution when attempting to extrapolate to whole cells and organisms. Firstly, the cell has a supplementary antioxidant capacity which can handle H_2_O_2_ efflux from the mitochondria and thus limit oxidative stress over a larger range of temperatures than the isolated mitochondria (Iftikar and Hickey, 2013). Second, the animal may adjust its behavior or physiology by reducing its activity or even undergoing metabolic depression (e.g., aestivation) when environmental resources become limited in order to alleviate the thermal change in ATP production efficiency (Bishop and Brand, 2000; Kayes et al., 2009).

## Conclusion

All of these results suggest that *B. bufo* would have to allocate extra energy to maintain ATP production and liver metabolism and to alleviate oxidative stress in order to survive an extremely hot-weather event. In addition, high temperatures will also induce behavioral adjustments that often result to restricting activity and foraging time (Kearney et al., 2009) or restricting space usage (Sears et al., 2016). Such lower capacity to obtain food together with the accelerating metabolic costs with a lower allocation of ATP to performances might lead to a “metabolic meltdown” of ectotherms for the next decades (Huey and Kingsolver, 2019). Nevertheless, the thermal decrease in coupling efficiency (ATP/O) and increase in ROS release can also trigger specific intracellular signaling pathways, which would contribute to the cellular energy metabolism adaptation and subsequent normalization of the initial intracellular perturbation (Seebacher et al., 2010; Ristow and Schmeisser, 2011; Zhang et al., 2018). Such cellular responses may promote long-term resistance to oxidative or metabolic challenges, ultimately contributing to the emergence of new phenotypes (Marrot et al., 2017).

## Data Availability Statement

The datasets generated for this study are available on request to the corresponding author.

## Ethics Statement

The animal study was reviewed and approved by Prefecture du Rhône and the French Ministry of Agriculture (DSV permit no. 692661232).

## Author Contributions

DR and YV contributed to the conception of the study and the design of the work, the validation and statistic of data, and the writing and editing of the manuscript. DR took charge of the acquisition and analysis of data.

## Conflict of Interest

The authors declare that the research was conducted in the absence of any commercial or financial relationships that could be construed as a potential conflict of interest.
